# Metabolomic Cerebrospinal Fluid Biomarkers for the Diagnosis of Atypical Parkinsonian Syndromes

**DOI:** 10.3390/ijms27073270

**Published:** 2026-04-03

**Authors:** Lan Ye, Florian Wegner, Nadine J. Smandzich, Olivia Rudtke, Gül Deniz Efe, Matthias Höllerhage, Ishana Viktoria Schneidereit, Stephan Greten, Sven Schuchardt, Martin Klietz

**Affiliations:** 1 Department of Neurology, Hannover Medical School, Carl-Neuberg-Straße 1, 30625 Hannover, Germany; wegner.florian@mh-hannover.de (F.W.); smandzich.nadine@mh-hannover.de (N.J.S.); rudtke.olivia@mh-hannover.de (O.R.); guel.d.efe@stud.mh-hannover.de (G.D.E.); hoellerhage.matthias@mh-hannover.de (M.H.); schneidereit.ishana@mh-hannover.de (I.V.S.); greten.stephan@mh-hannover.de (S.G.); klietz.martin@mh-hannover.de (M.K.); 2 Systems Neuroscience Hannover (ZSN), 30625 Hannover, Germany; 3 Department of Bio- and Environmental Analytics, Fraunhofer Institute for Toxicology and Experimental Medicine (ITEM), 30625 Hannover, Germany; sven.schuchardt@item.fraunhofer.de

**Keywords:** progressive supranuclear palsy, multiple system atrophy, metabolomics, CSF biomarkers

## Abstract

Diagnosis of atypical parkinsonian syndromes (APS), including progressive supranuclear palsy (PSP) and multiple system atrophy (MSA), rely on clinical criteria that often result in misclassification or delayed confirmation. Cerebrospinal fluid (CSF) metabolomics offers the potential to identify disease-specific biochemical “fingerprints”. The aim of the study is to identify CSF metabolomic biomarkers that distinguish PSP and MSA from each other and from non-neurodegenerative controls. Targeted mass spectrometry-based metabolomics was performed on CSF samples from 30 patients with MSA, 41 with PSP, and 30 age- and sex-matched non-neurodegenerative controls. Global metabolomic profiles showed no clear group separation. Both PSP and MSA showed elevated gut-derived metabolites p-cresyl sulfate and deoxycholic acid versus controls. In PSP, decreased cortisone and increased hexosylceramide d18:1/24:1 were observed, whereas in MSA, dihydroxyphenylalanine was elevated alongside homoarginine and creatinine. In the direct comparison of APS, levels of α-aminoadipic acid were increased in PSP compared to MSA. Pathway analysis highlighted disrupted glycerophospholipid metabolism in both APS disorders. Distinct metabolite panels mainly combining membrane-associated lipids, gut-derived and neurotransmitter-related metabolites demonstrated high diagnostic accuracy for distinguishing PSP and MSA from control groups (AUC = 0.95 for PSP and AUC = 0.98 for MSA), while a separate panel showed moderate performance in differentiating PSP from MSA (AUC = 0.85). Distinct but partially overlapping CSF metabolomic profiles characterize PSP and MSA. These metabolomic fingerprints highlight gut–brain axis involvement, alterations in cell membrane-related lipid metabolism, and disease-specific changes in neurotransmitter-related metabolites. Further, a panel of these metabolites showed strong potential as diagnostic biomarkers.

## 1. Introduction

Atypical parkinsonian syndromes (APS), including progressive supranuclear palsy (PSP) and multiple system atrophy (MSA), are rapidly progressive neurodegenerative diseases with substantial clinical overlap. PSP is characterized by supranuclear gaze palsy, early postural instability and cognitive impairments [1,2] while MSA presents with autonomic failure, levodopa unresponsive Parkinsonism, and/or cerebellar ataxia [3,4].

Despite advances in clinical criteria, APS diagnosis in routine practice remains challenging because of heterogeneity and overlap between PSP, MSA, and Parkinson’s disease (PD) [5,6]. Diagnostic uncertainty is common, and a definite diagnosis is often established only in advanced disease stages or even post-mortem [5,7,8]. Although no disease-modifying therapy is approved for PD or APS, the heterogeneity of Parkinsonism calls for precision medicine in which the various symptomatic therapies are individually tailored to match each patient’s priorities and needs for motor and non-motor complaints. This important development toward personalized precision medicine requires in turn an accurate diagnosis. Moreover, an accurate diagnosis enables a reliable prognosis and the appropriate inclusion in clinical trials.

Metabolomics provides a systematic assessment of metabolic changes and can generate biomarker signatures reflecting genetic, proteomic, environmental, and microbiome-related influences [9]. Several studies have explored metabolomic alterations in APS using plasma or serum [10,11,12]. Blood is an easily accessible biofluid, and a reliable serum/plasma biomarker would be very valuable in APS. However, considering the proximity of cerebrospinal fluid (CSF) to the central nervous system (CNS) and its reflection of the pathophysiological disease state [13], the investigation of metabolic changes in CSF allows a more direct assessment of the underlying neurodegenerative processes in APS, potentially leading to the identification of more specific and sensitive biomarkers. In general, CSF is regarded as a more precise indicator of CNS changes than other fluids, such as blood, saliva, and urine [14].

CSF metabolomics studies in APS remain limited, often due to small sample sizes or combined APS groupings despite distinct neuropathology. PSP is a four-repeat tauopathy with neuronal and glial pathology predominantly affecting basal ganglia, brainstem and cerebellar structures [15] while the hallmarks of MSA are oligodendroglial cytoplasmic inclusions rich in aggregated α-synuclein [16].

This study aims to decipher specific metabolomic fingerprints in CSF to enhance the diagnostic accuracy of PSP and MSA using a comparatively large and well-characterized study population. Additionally, these data may contribute to a better understanding of pathophysiological processes in APS and to the identification of potential targets for therapy.

## 2. Results

### 2.1. Demographic Data and Metabolomic CSF Profiles

Demographic and clinical characteristics of MSA, PSP and the sex- and age-matched control patients are presented in Table 1. A total of 499 metabolites (small molecules and lipids) were measured in CSF and no robust differences in the general metabolomic profiles among the three groups were identified (Figure S1). No clear sex-specific metabolomic differences were observed in either the PSP or MSA groups (Figure S2).

**Figure 1:**
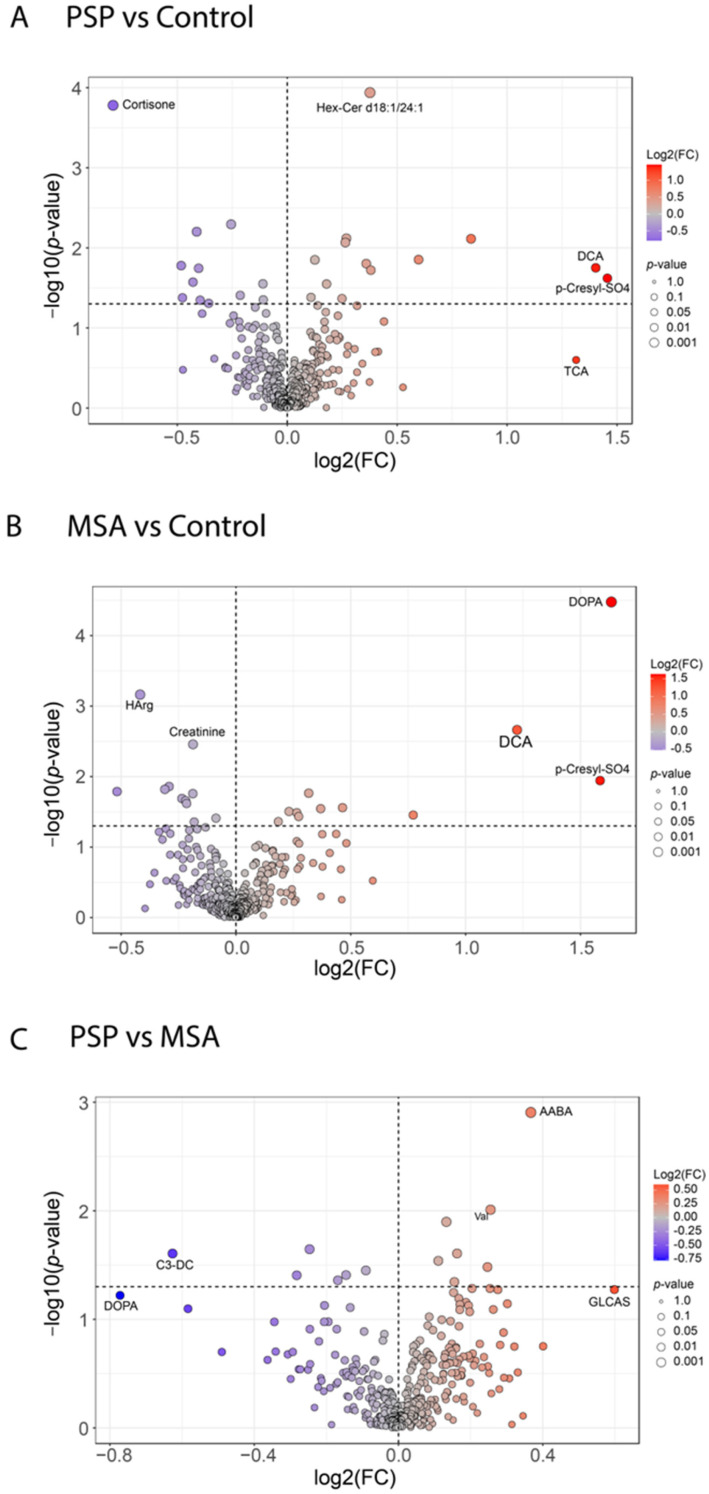
Metabolic differences among PSP, MSA and control groups. Volcano plots of CSF metabolite differences among PSP, MSA, and controls. Panels: (**A**), PSP vs. controls; (**B**), MSA vs. controls; (**C**), PSP vs. MSA. Each point represents a single metabolite. The x-axis shows the log_2_ fold change (FC), indicating the magnitude and direction of change in metabolite abundance between the two groups. Positive Iog_2_FC values indicate higher abundance in one group, while negative values indicate higher abundance in the other. The y-axis represents the negative log_10_ of the *p*-value, which reflects the statistical significance of the observed difference. Higher values on the y-axis correspond to lower *p*-values (more significant). Metabolites above the dashed horizontal line are considered significant (*p* < 0.05; i.e., −log_10_(*p*) > 1.301). Key metabolites with significant changes are highlighted with their names, including DOPA, DCA and p-cresyl-SO_4_ in (**A**), DCA and p-cresyl-SO_4_ in (**B**) as well as AABA (Alpha-Aminobutyric acid) in (**C**). Abbreviations: AABA, α-aminobutyric acid; C3-DC, malonylcarnitine; CSF, cerebrospinal fluid; DCA, deoxycholic acid; GLCAS, glycolithocholic acid sulfate; DOPA, dihydroxyphenylalanine; FC, fold change; HArg, Homoarginine; MSA, multiple system atrophy; p-cresyl-SO_4_, p-cresyl sulfate; PSP, progressive supranuclear palsy; TCA, taurocholic acid.

As shown in the volcano analysis (Figure 1A), in the PSP group compared to controls, p-cresyl sulfate (p-cresyl-SO_4_) and deoxycholic acid (DCA) were significantly elevated (log_2_ fold change > 1.25; *p* < 0.05), whereas hexosylceramide (Hex-Cer) d18:1/24:1 was slightly elevated (log_2_ fold change > 0 but <0.5; *p* < 0.05). Taurocholic acid (TCA) increased prominently but did not reach statistical significance (log_2_ fold change > 1.25; *p* > 0.05). A significant reduction in CSF cortisone was observed (log_2_ fold change < −0.5; *p* < 0.05).

In the MSA group versus controls (Figure 1B), p-cresyl-SO_4_ and DCA were also significantly elevated (log_2_ fold change > 1; *p* < 0.05). Moreover, dihydroxyphenylalanine (DOPA) was markedly increased (log_2_ fold change > 1.5; *p* < 0.05) and homoarginine (HArg) and creatinine were slightly decreased (log_2_ fold change < 0 but >−0.5; *p* < 0.05).

In the direct comparison between MSA and PSP cohorts (Figure 1C), levels of α-aminobutyric acid (AABA) and valine (Val) were gently elevated in PSP (log_2_ fold change > 0 but <0.04; *p* < 0.05), while levels of malonylcarnitine (C3-DC) were significantly reduced (log_2_ fold change < −0.6; *p* < 0.05) in PSP relative to MSA. DOPA was decreased but did not reach statistical significance (log_2_ fold change < −0.6; *p* > 0.05). Similarly, glycolithocholic acid sulfate (GLCAS), another type of bile acid similar to DCA and TCA, showed a noticeable increase but did not reach statistical significance (log_2_ fold change > 0.5; *p* > 0.05).

**Figure 2:**
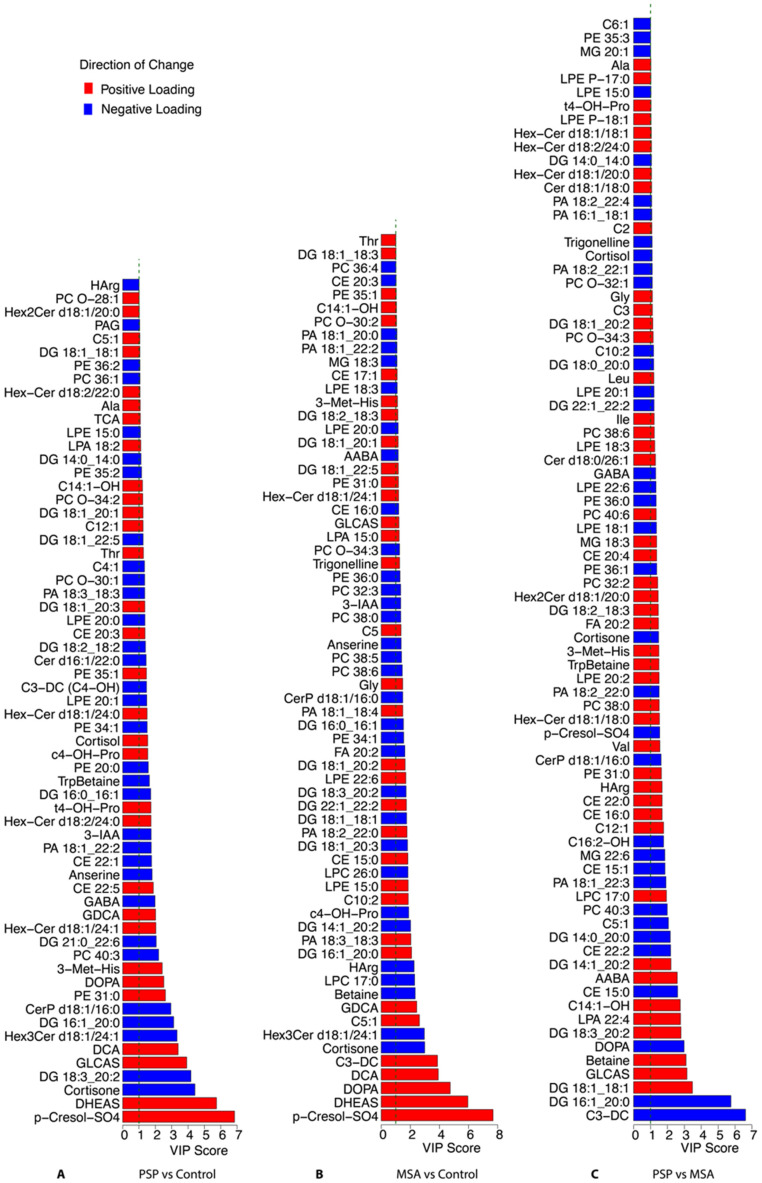
Metabolites with a VIP score component greater than 1 distinguishing PSP, MSA and controls in the partial least squares discriminant analysis. This plot ranks the metabolites based on their VIP scores, indicating their importance in distinguishing among PSP and controls. Panels: (**A**), PSP vs. controls; (**B**), MSA vs. controls; (**C**), PSP vs. MSA. The accompanying heatmap displays standardized (e.g., z-scored) relative abundance of these metabolites, with red indicating higher and blue indicating lower levels. Abbreviations: MSA: multiple system atrophy; PSP: progressive supranuclear palsy; VIP, variable importance in projection.

The PLS-DA model was further used to identify the metabolites that are most important for distinguishing between the groups (Figure 2); 60–90 metabolites in each comparison group reached a VIP value greater than one. These metabolic alterations identified in the volcano plot analysis were also captured within the PLS-DA model, supporting the consistency and robustness of the observed metabolic differences.

### 2.2. Metabolomic Pathways

**Figure 3:**
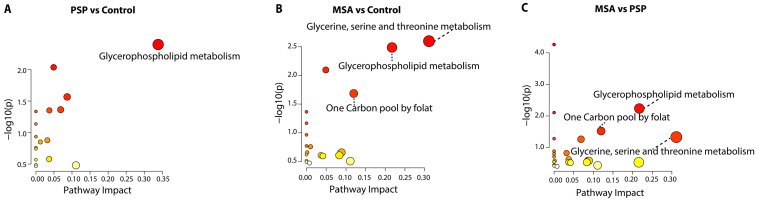
Pathway analysis revealing disease-associated metabolic alterations. Metabolites with a VIP score greater than 1 from Figure 2 were included for metabolic pathway analysis. Results are shown for (**A**) PSP vs. control, (**B**) MSA vs. control, and (**C**) MSA vs. PSP. The x-axis indicates pathway impact, and the y-axis shows statistical significance as −log_10_(*p*-value). Circle size is proportional to the number of metabolites involved in each pathway, and color intensity reflects the *p*-value, with darker red indicating lower *p*-values and brighter yellow indicating higher *p*-values.

As shown in Figure 3, disruptions in the glycerophospholipid metabolism pathway were observed in the CSF of patients with PSP compared to controls. Among the major classes of glycerophospholipids, phosphatidic acid (PA), phosphatidylcholine (PC) and phosphatidylethanolamine (PE) turned out to be the principally altered glycerophospholipids in PSP (Figure S3). The specific subtypes affected are listed in Table S1.

Similar disturbances in the glycerophospholipid metabolism pathway were also found in the CSF of patients with MSA compared to the controls, including alterations in PE and PC. Notably, the PCs associated with MSA largely differed from those seen in PSP (Table S1). Additionally, a decrease in 1-acyl-sn-glycero-3-phosphocholine, represented by lysophosphatidylcholine (LPC) 17:0, was observed. Beyond glycerophospholipid metabolism, MSA showed disruptions in the following two additional pathways: the one-carbon pool by folate, with elevated glycine and betaine, and glycine/serine/threonine metabolism, involving glycine, betaine, and L-threonine.

Comparison between MSA and PSP showed that glycerophospholipid, one-carbon, and glycine/serine/threonine metabolism were altered, consistent with the changes observed in MSA versus controls. However, in the glycerophospholipid metabolism, although the key metabolites remained PE, PC, and LPC, the most involved specific PEs and PCs were distinct despite some overlaps. Several metabolites showed consistent changes in both disease groups compared to controls: PE 35:1 was increased, while PA 18:1_22:2 and PA 18:3_18:3 were decreased in both PSP and MSA. PE 31:0 was elevated across all three comparisons, indicating an increase in both PSP and MSA, with a more pronounced elevation in PSP. PC O-34:3, PC 38:6, PC 38:0, and LPC 17:0 exhibited higher loadings in PSP compared to MSA, which aligns with their reduced levels in MSA relative to controls. In contrast, PC 40:3 was decreased in PSP against controls as well against MSA, indicating a PSP-specific reduction (Table S1).

### 2.3. The Potential Biomarkers to Discriminate MSA, PSP and Control

The ROC-based combinatorial analysis identified distinct biomarker panels for PSP and MSA. As detailed in Table 2, the panel distinguishing PSP from controls included gut-derived metabolites (p-cresyl-SO_4_ and DCA), glycerophospholipids (PE 20:0, PE 34:1, PE 35:2, PC O-28:1, and PC O-30:1), the sphingolipid Hex-Cer (d18:1/24:1), and cortisone, achieving an AUC of 0.95, with both sensitivity and specificity at 0.90.

The MSA panel shared gut-derived metabolites (p-cresyl-SO4 and DCA) but featured different glycerophospholipids (PC O-34:3, PC 38:6, PC 38:0, and LPC 17:0) and amino acid-related metabolites (threonine, betaine, and HArg), reaching an AUC of 0.98, with 0.97 sensitivity and 0.90 specificity.

To distinguish PSP from MSA, the panel included AABA, C3-DC, and glycerophospholipids (PE 35:3, PE 36:1, PC 32:2, PC 40:3, PC 38:6, PC O-34:3, and PC 40:6). This panel demonstrated an AUC of 0.85, with sensitivity and specificity values of 0.83 and 0.73, respectively. An additional inclusion of PE 36:0 resulted in an unchanged AUC of 0.85; however, sensitivity declined to 0.70, while specificity improved to 0.85.

## 3. Discussion

Our results suggest that the CSF metabolomic profiles of patients with MSA and PSP are distinct from those without neurodegeneration. Several metabolites were altered in the CSF of patients with MSA and PSP, including gut-derived metabolites, glycerophospholipids, sphingolipids, and transmitter-related metabolites. These metabolites may serve as potential biomarkers for these diseases and may facilitate the development of new diagnostic and therapeutic strategies. Despite the distinct pathological features of MSA (α-synucleinopathy) and PSP (4-repeat tauopathy) [15,16], their general CSF metabolomic profiles showed only subtle differences. We assume that most subtle metabolic changes related to the underlying proteinopathy were largely masked by severe neurodegeneration.

P-cresyl-SO_4_ and DCA were identified in both biomarker panels and showed significantly elevated levels in the CSF of patients with MSA and PSP. P-cresyl-SO_4_ is a sulfate conjugate of the uremic toxin p-cresyl and formed by bacterial fermentation of proteins in the large intestine [17]. P-cresyl-SO_4_ was found to be elevated in the serum [18] and in the CSF [19,20] of PD patients, and more enriched in the plasma of PD patients with REM sleep behavior disorder than in those without [21]. Taken together with our findings, these reports support the involvement of p-cresyl-SO_4_ in the pathogenesis of Parkinsonism and suggest that the gut microbiome may play a role in this group of diseases. Over the past decade, numerous studies have illuminated the interplay between gut and brain in Parkinsonism [22,23].

DCA, a bile acid involved in emulsifying dietary fats in the intestine, was also significantly higher in the CSF of both PSP and MSA patients. DCA could not only disrupt cell membranes in adipocytes but also lead to peripheral nerve damage in rats [24]. Although its direct effects on CNS remain unreported, its elevation in CSF further supports the involvement of gut-derived metabolites in APS. Other two bile acids, taurocholic acid (TCA) and glucolithocholic acid sulfate (GLCAS), also showed elevated trends in the CSF of PSP patients compared to the control and compared to MSA, implying a shared but distinct involvement of gut-derived metabolites in PSP and MSA.

Glycerophospholipids are key components of the biomarker panels, with distinct species characterizing PSP and MSA profiles. They are the main constituents of neuronal and glial membranes and include several classes, such as phosphatidic acid (PA), phosphatidylcholine (PC), phosphatidylethanolamine (PE), phosphatidylserine, phosphatidylglycerol, phosphatidylinositol, cardiolipin, lysophosphatidic acid (LPA) and lysobisphosphatidic acid. Among them, PC O-28:1, PC O-30:1, PE 34:1 and PE 35:2 were the key glycerophospholipids contributing to identifying PSP. PC is a fundamental membrane component and a source of choline [25], while PE is linked to protein biogenesis, oxidative phosphorylation, autophagy, membrane fusion, mitochondrial stability, and serves as a precursor for other lipids [26]. In MSA, PC O-34:3, PC 38:6, PC 38:0 and LPC 17:0 are the most essential glycerophospholipids. LPC 17:0, derived from phosphatidylcholine, is a component of oxidized low-density lipoprotein and plays a role in atherosclerosis, and cell signaling, etc. [27]. Furthermore, PE 35:3, PE 36:0, PE 36:1, PC 32:2, PC 40:3, PC 38:6, PC O-34:3 and PC 40:6 could serve as biomarkers to distinguish PSP from MSA patients. Although the precise physiological distinctions among the various subtypes of glycerophospholipids remain poorly understood, disturbed glycerophospholipid metabolism in the CNS is related to oxidative stress, mitochondrial dysfunction, inflammation and the apoptosis of neurons [28], which could contribute to the neurodegeneration in both MSA and PSP. Similar involvement of the glycerophospholipid pathway has been reported to be associated with the progression of Alzheimer’s disease (AD) [29] and amyotrophic lateral sclerosis [30]. Interestingly, the glycerophospholipid metabolism could be modulated by the gut microbiome [31,32], suggesting an additional mechanism linking gut microbiome to neurodegeneration.

Hex-Cer(d18:1/24:1), a ceramide belonging to the sphingolipid class, is also important in the biomarker-panel for PSP. It was slightly increased in the CSF of PSP patients compared to controls. Sphingolipids, another important type of lipid found in cell membranes, contribute to cell signaling and recognition, serve as integral components of myelin, and act as intermediates in inflammatory pathways [33]. This increase was not observed in the CSF of MSA, suggesting a different lipidomic fingerprint between the two diseases. One possible explanation is that in PSP both neurons and glial cells are affected [34] while in MSA the primarily affected cells are oligodendrocytes [16], the membranes of neurons and oligodendrocytes as well as other glial cell types differ in structure, composition and function [35].

Moreover, we found an involvement of fatty acid (FA) metabolism in MSA. FA 14:0 and FA 18:0 were previously reported to decrease in the plasma of MSA patients [11]. Although we did not observe similar alterations of these fatty acids in the CSF of MSA patients, FA 20:2 exhibited a negative loading, supporting the role of FA metabolism in MSA, albeit with different metabolic fingerprints in CSF compared to plasma. Notably, no FA alteration was identified in the plasma of PSP patients [11], and we did not find any significant changes in FA levels in the CSF of our PSP patients either. Moreover, C3-DC, which reflects malonyl-CoA accumulation and inhibition of mitochondrial FA β-oxidation [36], was higher in the CSF of MSA patients compared to PSP and could serve as a biomarker to distinguish the two diseases.

Neurotransmitter-related metabolites also differed between PSP and MSA. AABA, an isomer of GABA, was slightly elevated in the CSF of PSP relative to MSA and could be used to distinguish PSP from MSA in the biomarker panel. Recent studies suggest that AABA and the serum GABA/AABA ratio may serve as potential biomarkers for both aging and physical function [37]. In addition, valine was mildly elevated in the CSF of PSP compared to MSA. Among the three branched-chain amino acids, valine is the only one that supports the synthesis of vesicular glutamate, a key neurotransmitter involved in excitatory signaling [38]. Moreover, dehydroepiandrosterone sulfate (DHEAS), a neurosteroid that modulate GABA A receptor, NMDA receptor, and the sigma subtype 1 receptor [39], was reported to be significantly increased in the plasma of MSA patients and could serve as a biomarker to separate MSA from PSP with an AUC of 0.79 [11]. Similarly, in our study, DHEAS showed positive loadings in the CSF of MSA patients compared to those without neurodegeneration. However, in the CSF of our PSP patients DHEAS also had a similar tendency, which contrasts with a previous report [11]. Furthermore, lower plasma levels of DHEAS have been considered as risk factors for AD [40]. The shared involvement of DHEAS across the three neurodegenerative diseases but with opposite directional changes address distinct pathophysiological roles. Glycine, a major inhibitory neurotransmitter [41], was increased in the CSF of MSA patients. Glycine inhibitors as PD therapy are under development [42]. Betaine, a derivative of glycine, has been shown to positively correlate with cognitive performance in PD patients [43], and was previously reported to be decreased in the plasma of MSA patients [11]. These reports align with our CSF findings. Moreover, it has been reported that dietary threonine reduced GABA levels, weakened metabotropic GABA responses in a subset of ellipsoid body neurons, and ameliorated memory deficits in plasticity mutants [44]. This is also consistent with our finding that threonine levels were increased in the CSF of MSA patients. Both betaine and threonine were identified as potential CSF biomarkers capable of distinguishing MSA patients from age- and sex-matched controls.

Other amino acid derivatives, including HArg and creatinine, were slightly decreased in the CSF of MSA patients. HArg is a natural substrate of nitric oxide production. Creatinine is the end product of creatine and creatine phosphate metabolism. Both metabolites are directly or indirectly related to L-arginine metabolism [45,46]. Lower levels of HArg and creatinine increased the risk of stroke [46,47], and creatinine has been reported to be decreased in the CSF of PD patients [48,49]. Moreover, creatine, creatinine and arginine were found to be increased in the urine and plasma of PD patients [50]. One possible explanation of the special association between creatinine and PD is that creatinine can be degraded in the gastrointestinal tract by gut bacteria and utilized as a nitrogen source, a process linked to constipation in PD patients [51]. This may help explain our observation in MSA, where constipation is also common. The co-decrease in HArg and creatine may reflect disturbed arginine-related metabolism in MSA.

Cortisone was significantly decreased in the CSF of patients with PSP and was identified as a potential biomarker. Although cortisone itself is biologically inactive, it can be converted to cortisol via 11β-hydroxysteroid dehydrogenase enzymes in liver, kidney, brain, etc. The underlying cause of its marked reduction in PSP remains unclear, but it may reflect alterations in steroid metabolism or neuroendocrine regulation.

DOPA was found to be significantly increased in the CSF of MSA patients. In patients with PSP, the analysis also showed a tendency of DOPA increase, but not as significant as in MSA. These findings contrast with previous reports of reduced L-DOPA levels in the CSF of patients with synucleinopathies, including PD and MSA [52], but are more likely attributable to medication effects, as we did not exclude patients who were undergoing L-DOPA therapy. The average L-DOPA dose in MSA patients in this study was 430.0 ± 378.5 mg while it was slightly lower in PSP with 376.8 ± 352.6 mg. In our previous study, it was shown that 77.5% MSA patients were treated with L-DOPA [53], while 67.7% PSP patients received L-DOPA [54], despite the overall only marginal effect of L-DOPA on these patients [55]. In Goldstein et al., one of the patients on L-DOPA also had a very high CSF concentration of L-DOPA [52]. Therefore, the increased DOPA in our MSA patients is most likely a result of the pharmaceutical effect rather than a biological process. However, because our assay measured total DOPA (L-DOPA, D-DOPA and DL-DOPA), we could not exclude contributions from the other two non-pharmacological isoforms.

Beyond the shared disruption in glycerophospholipid metabolism in MSA and PSP, two additional metabolic pathways—one-carbon metabolism and glycine/serine/threonine metabolism—were uniquely altered in MSA. Disruptions in one-carbon metabolism and elevated homocysteine were previously implicated in the development of dementia associated with AD and PD [43]. While L-DOPA treatment could interrupt the one-carbon metabolism [43], we could not rule out its influence here, as 70% of our MSA patients were receiving L-DOPA treatment. The same could apply to glycine, serine and threonine metabolism, since the metabolites betaine and glycine in this pathway are the same as those involved in the one-carbon pool by folate pathway. However, similar L-DOPA exposure in PSP suggests these disturbances are disease-specific rather than drug-related.

Several metabolites previously reported to be associated with MSA and PSP were not confirmed or tested in our study. L-citrulline [56] and eicosapentaenoic acid (icosapent) [57] were reported to be increased in the CSF of MSA patients. However, these observations were not replicated in our cohort. A possible explanation for this discrepancy may lie in the differences between the control groups. Other metabolites previously reported to be significantly altered in the CSF of MSA patients, such as norepinephrine, coenzyme Q10, lactic acid, etc. [13], were not tested using our analytical method.

Understanding the metabolomic changes between patients with MSA and PSP is crucial for advancing our knowledge of these diseases. By further investigating longitudinal changes in metabolomics, we may uncover early biomarkers for the prodromal stages of these conditions, enabling earlier diagnosis and potentially the development of more effective treatments and prevention strategies.

This study has several limitations. First, it was conducted within a single cohort at one university hospital; external validation in an independent cohort will be necessary to confirm their robustness and generalizability of the study results, especially the performance of the diagnostic panel. Second, exclusion of patients treated with L-DOPA or other anti-parkinsonian medications was not possible, and pharmacological interactions therefore cannot be ruled out. Finally, clinical diagnoses were not histologically confirmed.

## 4. Materials and Methods

### 4.1. Study Participants

Ethical approval was obtained from the local Ethics Committee at Hannover Medical School (No. 8666_Bo_K_2019) and was conducted in accordance with the Declaration of Helsinki. All patients gave their written informed consent to participate in this study. Clinical data included sex, age at examination, comorbidities, disease duration, Hoehn and Yahr stage, phenotype, unified Parkinson’s disease rating scale part III (UPDRS III), levodopa dose (LD), levodopa equivalent daily doses (LEDD) [58], progressive supranuclear palsy rating scale (PSPRS) for PSP patients and unified multiple system atrophy rating scale part I and part II (UMSARS I&II) for MSA patients. CSF samples were taken by standardized lumbar puncture from 30 patients diagnosed with MSA, 41 with PSP and 30 control patients. Diagnoses were determined by a movement disorder specialist according to Movement Disorders Society diagnostic criteria for PSP [2] and MSA [3]. The control group consisted of individuals who received neurological diagnostic workup, including lumbar puncture, in the neurology department. These participants did not present with neurodegenerative diseases, autoimmune disorders, infections, neoplasms, or cerebrovascular events. However, individuals exhibiting nonspecific neurological symptoms, such as headache, paresthesia, or muscle pain, as well as those diagnosed with idiopathic polyneuropathy or idiopathic intracranial hypertension, were eligible for inclusion.

### 4.2. CSF Collection

The procedure of lumbar puncture was performed in routine neurological care on a ward of the Department of Neurology. All samples were obtained between 9 a.m. and 2 p.m. Patients were not in fasting condition while CSF collection was performed. CSF samples were collected into polypropylene tubes. These were transferred immediately to the laboratory where probes were centrifuged at 2000× *g* for 10 min at 4 °C. CSF without cell components was collected and transferred on ice to the Hannover Unified Biobank (HUB) [59]. At HUB, the probes were manually divided into 500 μL aliquots and stored in Matrix™ ScrewTop V-Bottom Tubes (Virgin Class VI Medical Grade Polypropylene, Rochester, NY, USA) in the vapor phase of liquid nitrogen (≤−190 °C) until further analysis.

### 4.3. Targeted Metabolomic Analysis

As previously reported [60], metabolomic profiling was performed using the MxP^®^ Quant 500 XL kit (Biocrates Life Sciences AG, Innsbruck, Austria) according to the manufacturer’s instructions (user manual v4-2024). Briefly, 10 µL of CSF per sample was processed on 96-well plates and analyzed on a 5500 QTRAP mass spectrometer (SCIEX, Darmstadt, Germany) equipped with electrospray ionization and coupled to an Agilent 1290 Infinity II UHPLC system (Agilent Technologies, Santa Clara, CA, USA). This kit enables the simultaneous quantification of 1019 metabolites spanning 15 small-molecule and 25 lipid classes by combining LC–MS/MS for small molecules and FIA–MS/MS for lipids in multiple reaction monitoring (MRM) mode. Data acquisition and peak integration were performed using SCIEX Analyst/MultiQuant software (v1.7.2) and processed in Biocrates WebIDQ (v.DB135-864) for analyte identification, quality-control assessment, and calculation of absolute concentrations. Analytical runs were accepted only if all kit quality-control criteria met the manufacturer’s specifications. At the metabolite level, only analytes with valid measurements in ≥80% of samples within each study group and passing all kit status flags were retained for downstream analyses. Values below the limit of detection (LOD) were imputed as LOD/2, whereas values between the LOD and the lower limit of quantification (LLOQ) were retained and flagged.

### 4.4. Statistical Analysis

The analysis was performed using software R Studio (Version 2024.12.1) with the MetaboAnalyst package as well as the website of the MetaboAnalyst 6.0 [61]. The normality of numeric demographic and clinical data was assessed using the Shapiro–Wilk test. For comparisons among three groups, one-way ANOVA was applied to normally distributed variables, while the Kruskal–Wallis test was used for non-normally distributed data. For two-group comparisons, Welch’s *t*-test was used for normally distributed data, and the Mann–Whitney U test for non-normal distributions. The chi-square test was performed to compare proportions for categorical variables. To compare the overall metabolome profile across the three groups (MSA, PSP, and controls), Principal Component Analysis (PCA) was employed. The volcano analysis was used to identify significant changes in the metabolic profiles across the three groups. Metabolites showing a *p* < 0.05 were considered significant. Partial least squares discriminant analysis (PLS-DA) was performed to identify metabolites that discriminate between different groups. Variable importance in projection (VIP) scores was computed to evaluate the contribution of each metabolite to the classification of the disease groups by the PLS-DA latent variables. All metabolites with a VIP value larger than 1 were determined to be a candidate biomarker and were involved in the metabolic pathway analysis. The key metabolites were further selected based on the following two complementary approaches: (1) metabolites showing significant changes in volcano analysis (*p* < 0.05), and (2) key metabolites identified in the metabolic pathway analysis. The diagnostic performance of metabolite combinations was calculated using multivariable logistic regression and Receiver Operating Characteristic (ROC) analysis for the following three binary classifications: MSA vs. PSP, MSA vs. controls, and PSP vs. controls. For each binary comparison, logistic regression models were fitted and predicted probabilities were used to generate ROC curves and calculate the area under the curve (AUC). The optimal cutoff was defined by the Youden index, and sensitivity and specificity at this threshold were reported.

## 5. Conclusions

We found a distinct metabolomic fingerprint between MSA and PSP albeit a marked overlap and identified biomarkers in the CSF that could distinguish PSP and MSA patients from age- and sex-matched controls with high sensitivity and specificity. A CSF biomarker panel comprising gut-derived metabolites p-cresyl-SO4 and DCA, specific subtypes of glycerophospholipids and sphingolipids, which are both crucial for cell membrane structure and function, effectively distinguished patients with PSP from age- and sex-matched controls. Meanwhile, a separate panel including the same gut-derived metabolites, but other specific subtypes of glycerophospholipids, together with neurotransmitter-related metabolites, showed strong discriminatory power for identifying MSA patients compared to controls.

Our data support the hypothesis that gut microbiome may contribute to the pathogenesis of MSA and PSP. This is indicated by elevated levels of p-cresyl-SO4 and increased concentrations of DCA, alongside similar trends observed for other bile acids. Additionally, the reduction in creatinine in MSA, which is related with microbial degradation in the gastrointestinal tract, further reinforces this connection. Notably, the observed disturbances in glycerophospholipid metabolism may also be influenced by microbial activity, suggesting a broader impact of the gut–brain axis on lipid homeostasis and neurodegenerative processes.

Beyond the distinct glycerophospholipid and sphingolipid changes associated with the pathogenesis of MSA and PSP, our results indicate that a specific fatty acid metabolism is uniquely involved in MSA. This is evident from the decrease in fatty acid levels and the increase in C3-DC, whose elevation reflects upstream changes in fatty acid metabolism in the CSF of MSA patients. Additionally, the varying degrees of involvement of neurotransmitter-related metabolites in MSA and PSP are also important for distinguishing between these two diseases.

## Figures and Tables

**Table 1:** Demographic and clinical characteristics of the study participants.

	MSA *n* = 30	PSP *n* = 41	Control *n* = 30	*p*-Value
Age (years)	61.5 ± 9.4	65.8 ± 8.4	62.4 ± 9.4	F(2, 147) = 2.25, *p* = 0.111
Sex (men)	17/30 (56.7%)	17/41 (41.5%)	13/30 (43.3%)	χ^2^(2)= 1.7852, *p* = 0.4096
Comorbidities	3.5 ± 2.7	4.8 ± 3.1	4.7 ± 4.2	χ^2^(2) = 2.45, *p* = 0.15
Hoehn and Yahr stage	3.5 ± 0.7 (*n* = 22)	3.2 ± 0.9 (*n* = 34)	/	W = 437, *p* = 0.2614
Disease duration (years)	3.7 ± 2.4 (*n* = 26)	2.9 ± 1.6 (*n* = 34)	/	W = 518.5, *p* = 0.2486
UPDRS III	42.1 ± 15.2 (*n* = 26)	33.3 ± 15.5 (*n* = 39)	/	t = 2.283, df = 54.441, *p* = 0.02636 *
LD (mg)	430.0 ± 378.5	376.8 ± 352.6	/	W = 656.0, *p* = 0.6322
LEDD (mg)	539.4 ± 451.5	458.0 ± 396.0	/	W = 675.0, *p* = 0.4861
Phenotype	MSA-C (*n* = 14, 46.6%) MSA-P (*n* = 16, 53.3%)	PSP-RS (*n* = 39, 95.1%) PSP-P (*n* = 1, 2.4%) PSP-CBS (*n* = 1, 2.4%)	/	/
UMSARS I and II	42.5 ± 11.8 (*n* = 20)	/	/	/
PSPRS	/	32.9 ± 13.6 (*n* = 35)	/	/

The Kruskal–Wallis test was used to compare the age among the three groups while the chi-squared test was performed to compare the distribution of the sex and the number of comorbidities. Mann–Whitney U test was applied to compare the Hoehn and Yahn stage and the disease duration between PSP and MSA while Welch’s *t*-test to compare the UPDRS III between them. * is considered to be significant. Abbreviations: LD: levodopa dose; LEDD: levodopa equivalent daily doses; MSA: multiple system atrophy; PSP: progressive supranuclear palsy; PSPRS: progressive supranuclear palsy rating scale; UMSARS: unified multiple system atrophy rating scale; UPDRS III: unified Parkinson’s disease rating scale part III. *p* < 0.05 considered to be statistically significant.

**Table 2:** The sensitivity and specificity of particular biomarker panels.

Group	The Panel of Metabolites	AUC	Sensitivity	Specificity
PSP vs. Control	p-cresyl-SO_4_ + DCA + Cortisone + Hex-Cer(d18.1:24:1) + PE 20:0 + PE 34:1 + PE 35:2 + PC O-28:1 + PC O-30:1	0.95	0.90	0.90
MSA vs. Control	p-cresyl-SO_4_ + DCA + HArg + Betaine + Threonine + PC O-34.3 + PC 38:6 + PC 38:0 + LPC 17:0	0.98	0.97	0.90
PSP vs. MSA	AABA + C3-DC + PE 35:3 + PE 36:1 + PC 32:2 + PC 40:3 + PC 38:6 + PC O-34:3 + PC 40:6	0.85	0.83	0.73
	AABA + C3-DC + PE 35:3 + PE36:0 + PE 36:1 + PC 32:2 + PC 40:3 + PC 38:6 + PC O-34:3 + PC 40:6	0.85	0.70	0.85

Abbreviations: AABA: α-aminoadipic acid; C3-DC: malonylcarnitine; DCA: deoxycholic acid; Hex-Cer: hexosylceramide; LPC: lysophosphatidylcholine; MSA: multiple system atrophy; PC: phosphatidylcholine; p-cresyl-SO_4_: p-cresyl sulfate; PE: phosphatidylethanolamine; PSP: progressive supranuclear palsy. Note: lipids are reported as class abbreviation followed by total carbon:number of double bonds (e.g., PC 34:1). Ether designations are indicated with O- for alkyl ether.

## Data Availability

The data that support the findings of this study are available from the corresponding author upon reasonable request.

## Supplementary Materials

The following supporting information can be downloaded at https://www.mdpi.com/article/10.3390/ijms27073270/s1.

## Abbreviations

The following abbreviations are used in this manuscript:

AABA	α-Aminobutyric acid
AD	Alzheimer’s disease
APS	Atypical Parkinsonian syndromes
AUC	Area under the curve
C3-DC	Malonylcanitine
CNS	Central nervous system
CSF	Cerebrospinal fluid
DCA	Deoxycholic acid
DHEAS	Dehydroepiandrosterone sulfate
DOPA	Dihydroxyphenylalanine
FA	Fatty acid
GABA	ɣ-Aminobutyric acid
GLCAS	Glycolithocholic acid sulfate
HArg	Homoarigine
Hex-Cer	Hexosylceramide
LD	Levodopa dose
LEDD	Levodopa equivalent daily dose
LPA	Lysophosphatidic acid
LPC	Lysphosphatidylcholine
MSA	Multiple system atrophy
PA	Phosphatidic acid
PC	Phosphatidylcholine
PE	Phosphatidylethanolamine
PD	Parkinson’s disease
PCA	Principal Component Analysis
PLS-DA	Partial least squares discriminant analysis
p-cresyl-SO_4_	p-cresyl sulfate
PSP	Progressive supranuclear palsy
PSPRS	Progressive supranuclear palsy rating scale
ROC	Receiver Operating Characteristic
TCA	Taurocholic acid
UMSARS I&II	Unified multiple system atrophy rating scale, part I and II
UPDRS III	Unified Parkinson’s disease rating scale, part III
VIP	Variable importance in projection
