# Supplementary material for: Metabolomic Cerebrospinal Fluid Biomarkers for the Diagnosis of Atypical Parkinsonian Syndromes
Source: Int J Mol Sci. 2026 Apr 3;27(7):3270. doi: 10.3390/ijms27073270 (PMC13073110; doi:10.3390/ijms27073270)
Supplement: Supplementary file 1 [file ijms-27-03270-s001.zip › ijms-4199570-supplementary.pdf]

Supplementary Methods

Table S1. Comparison of metabolites involved in the glycerophospholipid metabolism pathway between study groups.

	PSP vs Control		MSA vs Control		PSP vs MSA	
	↑	↓	↑	↓	↑	↓
PE	PE31:0***	PE20:0	PE 31:0***	PE 34:1	PE 31:0***	PE 35:3
	PE 35:1**	PE34:1	PE 35:1**	PE 36:0*		PE 36:0*
		PE35:2				PE 36:1
		PE 36:2				
PC	PC O-28:1	PC 36:1	PC O-30:2	PC 32:3	PC 32:2	
	PC O-34:2	PC 40:3*		PC 36:4	PC 38:0*	PC 40:3*
		PC O-30:1		PC 38:0*	PC 38:6*	PC O-32:1
				PC 38:5	PC 40:6	
				PC 38:6*	PC O-34:3*	
				PC O-34:3*		
PA		PA 18:1_22:2**	PA 18:1_18:4	PA 18:1_20:0		PA 16:1_18:1
		PA 18:3_18:3**		PA 18:1_22:2**		PA 18:1_22:3
				PA 18:3_18:3**		PA 18:2_22:4
LPC				LPC 17:0*	LPC 17:0*	

*** Metabolites exist in all three compared groups; ** Metabolites exist in both MSA vs. controls and PSP vs. control group; * Metabolites exist either in both MSA vs. controls and PSP vs. MSA or in both PSP vs. controls and PSP vs. MSA. **Abbreviations:** LPC: lysophosphatidylcholine; MSA: multiple system atrophy; PA: phosphatidic acid; PC: phosphatidylcholine; PE: phosphatidylethanolamine; PG: phosphatidylglycerol; PS: phosphatidylserine; PSP: progressive supranuclear palsy. Note: Lipids are reported as class abbreviation followed by total carbon:number of double bonds (e.g., PC 34:1). Ether designations are indicated with O- for alkyl ether.

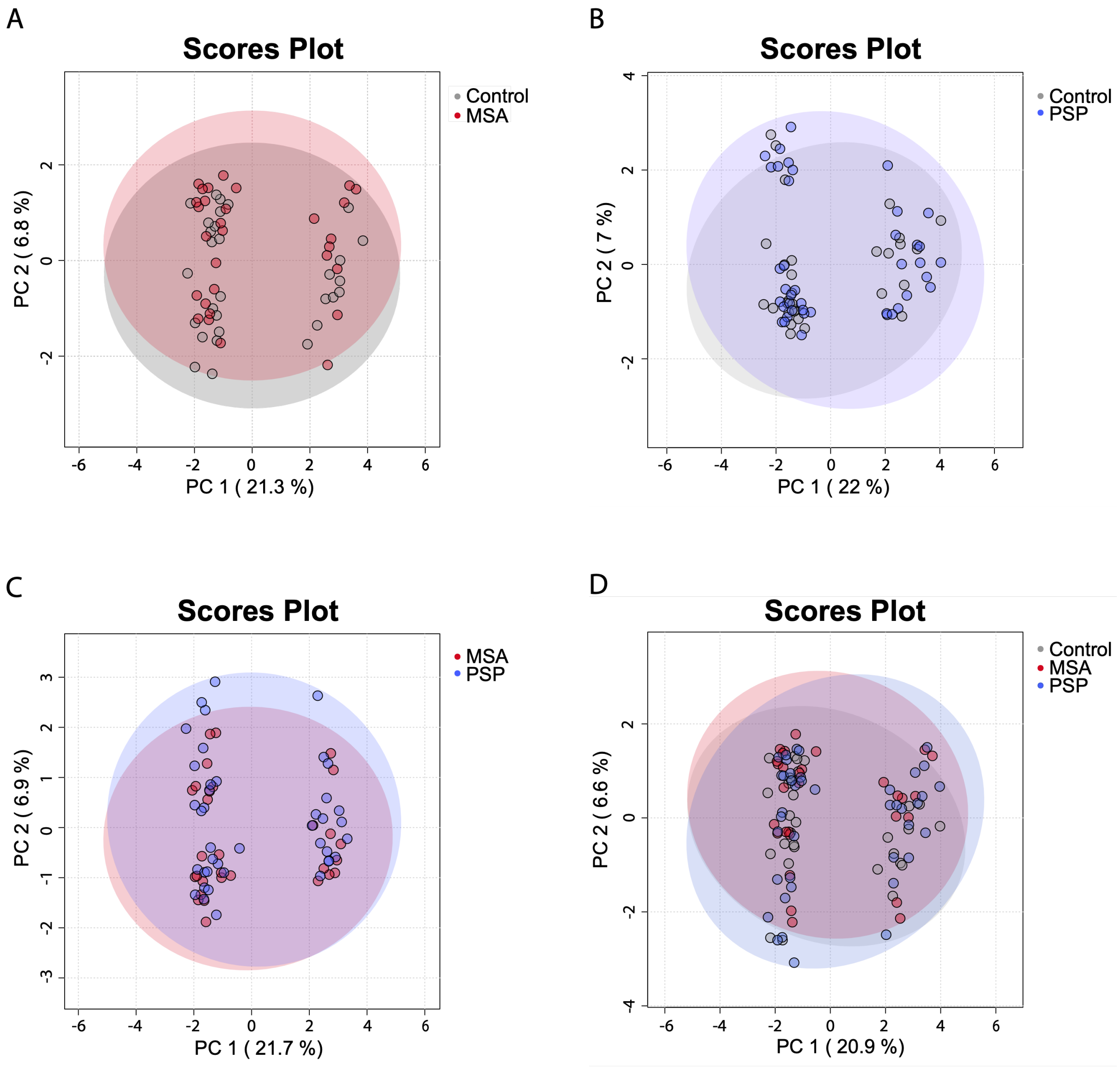

Figure S1. Comparison of the metabolomic profiles among MSA, PSP and controls with PCA. Each point represents a single CSF sample colored by group. Shaded ellipses denote the 95% confidence regions for each group. The percentage of variance explained by each principal component (PC1 and PC2) is indicated on the axes. A) control vs. MSA; B) controls vs. PSP; C) MSA vs. PSP; D) MSA vs. PSP vs. controls.

Abbreviations: CSF, cerebrospinal fluid; MSA: multiple system atrophy; PC, principal component; PCA: principal component analysis; PSP, progressive supranuclear palsy.

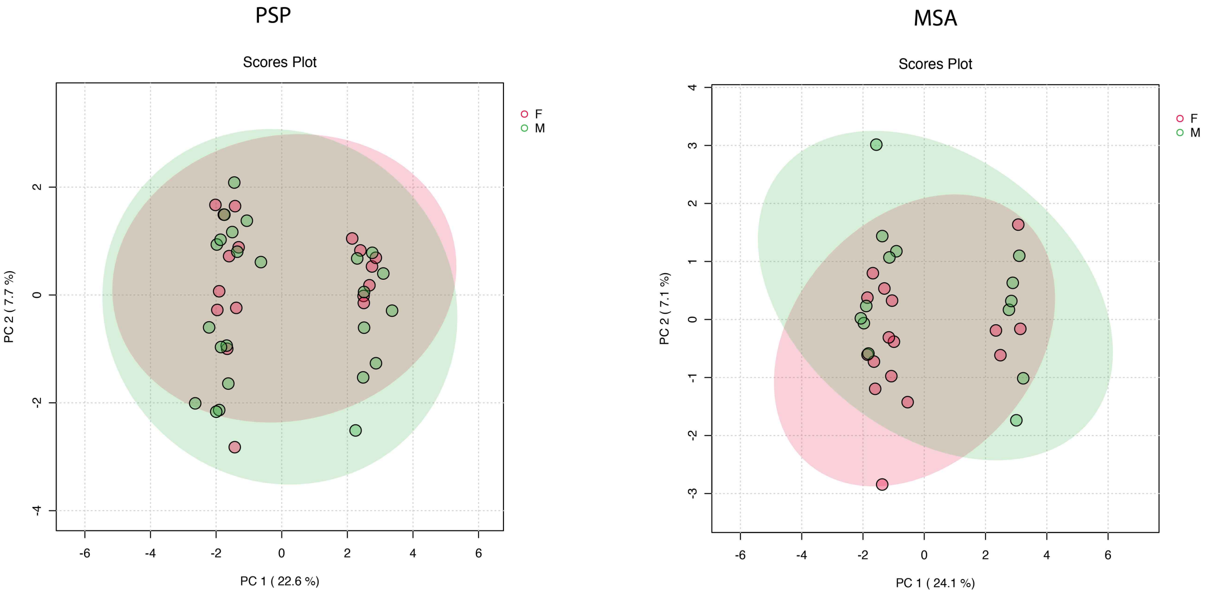

Figure S2. Comparison of the metabolomic profiles between male and female with PSP and MSA. Each point represents a single CSF sample colored by group of sex. Shaded ellipses denote the 95% confidence regions for each group. The percentage of variance explained by each principal component (PC1 and PC2) is indicated on the axes.

Abbreviations: F: female; M: male; MSA: multiple system atrophy; PC, principal component; PSP, progressive supranuclear palsy.

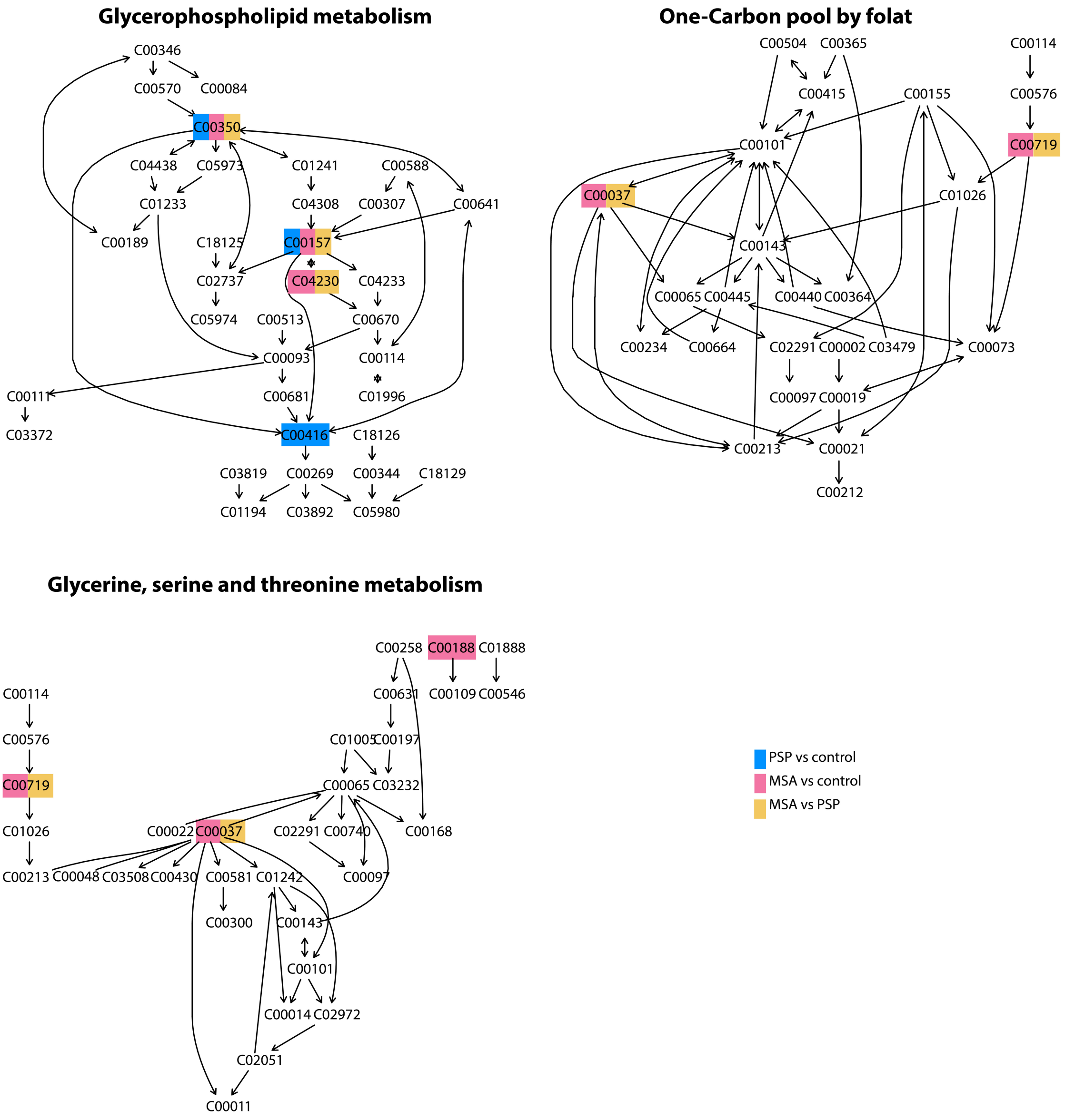

Figure S3. Pathway mapping of significantly altered metabolites in PSP and MSA.

Differential metabolites identified in the comparisons of PSP vs control, MSA vs control, and MSA vs PSP were mapped onto the metabolic pathways glycerophospholipid metabolism, one-carbon pool by folate, and glycine, serine and threonine metabolism. Metabolites are color-coded according to the comparison in which they were identified (blue, PSP vs control; pink, MSA vs control; yellow, MSA vs PSP), and multicolored boxes indicate metabolites shared across comparisons. Pathway connections are shown according

to KEGG annotations.

Highlighted metabolites include phosphatidylethanolamine (C00350), phosphatidylcholine (C00157), 1-acyl-sn-glycero-3-phosphocholine (C04230), phosphatidate (C00416), glycine (C00037), betaine (C00719), and L-threonine (C00188).

Abbreviations: MSA, multiple system atrophy; PSP, progressive supranuclear palsy.
